# Effects of a Gluteal Muscles Specific Exercise Program on the Vertical Jump

**DOI:** 10.3390/ijerph17155383

**Published:** 2020-07-27

**Authors:** Tomás Gallego-Izquierdo, Gerardo Vidal-Aragón, Pedro Calderón-Corrales, Álvaro Acuña, Alexander Achalandabaso-Ochoa, Agustín Aibar-Almazán, Antonio Martínez-Amat, Daniel Pecos-Martín

**Affiliations:** 1 Physiotherapy and Pain Group, Department of Physical Therapy, University of Alcala, 28801 Madrid, Spain; tomas.gallego@uah.es (T.G.-I.); daniel.pecos@uah.es (D.P.-M.); 2 Physical Therapist, Department of Physical Therapy, University of Alcala, 28801 Madrid, Spain; v.g.gerardo@gmail.com (G.V.-A.); pedrocalderon.fisio@gmail.com (P.C.-C.); alvaroab297@gmail.com (Á.A.); 3 Department of Health Sciences, Faculty of Health Sciences, University of Jaén, 23071 Jaén, Spain.; aaibar@ujaen.es (A.A.-A.); amamat@ujaen.es (A.M.-A.)

**Keywords:** squat, height, amateur athletes, training optimization, protocol

## Abstract

The vertical jump is a complex movement where many factors are involved in the final result. Currently, how a specific exercise program for gluteal muscles can affect the vertical jump is unknown. So, the aim of this study was to examine the effect of a specific exercise program for the gluteal muscles on a vertical jump. Forty-nine amateur athletes completed an 8-week program. The experimental group received a specific gluteal muscle training program in addition to their regular training routine, whereas the control group received their regular training routine. Jump height, flight time, speed and power were assessed (baseline, postintervention, and 4-week follow-up). Repeated-measures analyses of variance were conducted with ∝ ≤ 0.05. We calculated Eta squared effect sizes with 95% confidence intervals. Measurements at 8 weeks revealed significant increases in the experimental group compared to the control group for the values: jump height (*p* < 0.05) (experimental group = 17.15%; control group = 3.09%), flight time (*p* < 0.001) (experimental group = 7.98%; control group = 3.52%), speed (*p* < 0.01) (experimental group = 1.96%; control group = 1.83%) and power (*p* < 0.05) (experimental group = 4.43%; control group = 0.32%). However, at follow-up, these changes were not maintained. These data suggest that this specific training protocol for the gluteal muscles is effective in order to improve vertical jump performance in amateur athletes who use the vertical jump in their routine training habits.

## 1. Introduction

Vertical jump training has been studied for a long time because of its application in performance improvement and efficacy in sports, notably basketball, volleyball and football, among others [1,2]. It is a complex movement where simultaneous and coordinated activation of numerous muscle groups takes place [1,3]. Some of the most influential factors on vertical jump capability are, first, the power of the muscles involved and, secondly, the movement of the trunk and extremities, together with the size of the bony structures involved in a vertical jump [4,5,6]. A variety of research papers have referred to the summation effect of the anthropometric factors, coordination and anticipatory contraction of the muscle groups, and to the participation of the different joints of the limbs and trunk [7]. Muscles such as the quadriceps [8,9], the hamstrings [10] or the erector espinae [3,11] have been demonstrated to influence the vertical jump. It has been suggested that the gluteal muscles have a main role in squat jump due to their participation in the lateral pelvic stabilization in the frontal plane, in the case of the gluteus medius and maximus, and to their action on the impulse produced during the take-off phase, thanks to the great extensor component of the *gluteus maximus* [1]. However, the gluteus maximus is prone to weakness and inhibition due to prolonged sitting [12] and this could negatively affect sport performances. In addition, a decrease in the activity of the gluteus maximus would increase the reliance on the secondary hip extensor muscles (hamstrings), changing the motor pattern in order to keep the function and potentially leading to pain and strain injuries [13]. It has been hypothesized that specific training of this muscle group could increase the efficacy of the pelvic lever, giving it greater stability so the muscles can direct their power more effectively [1,14].

Assessment of the squat jump through a laboratory force plate is considered the “gold standard” [15]. Nevertheless, it has a high cost and low portability, which has contributed to the development of new more affordable and portable measurement systems, such as apps for mobile phones. This measurement system has been validated in children [16], athletes [17] and the elderly [18].

While strength training on the hamstrings [10] has been shown to have a beneficial impact on jumping, little is known about the impact of a gluteal muscle-specific exercise program on squat jump. The purpose of this study was to examine the influence of gluteal muscle-specific training on vertical jump. We hypothesized that an eight-week course of gluteal muscle-specific training would increase jump height, flight time, speed and power compared to a control group, and that any improvements would remain over time after the end of the intervention.

## 2. Materials and Methods

### 2.1. Study Design

An experimental research was carried out in order to determine gluteal muscle-specific training effects on vertical jump performance in amateur athletes who include the vertical jump in their sport activity. To address this, a simple blinded randomized design was used. The study was approved by the Research Ethics and the Animal Experimentation Committees of the University of Alcalá, according with the Declaration of Helsinki, good clinical practices, and applicable laws and regulations, and it was registered in the “Australian New Zealand Clinical Trials Registry” (ACTRN12618000751279p).

### 2.2. Participants

**Figure 1:**
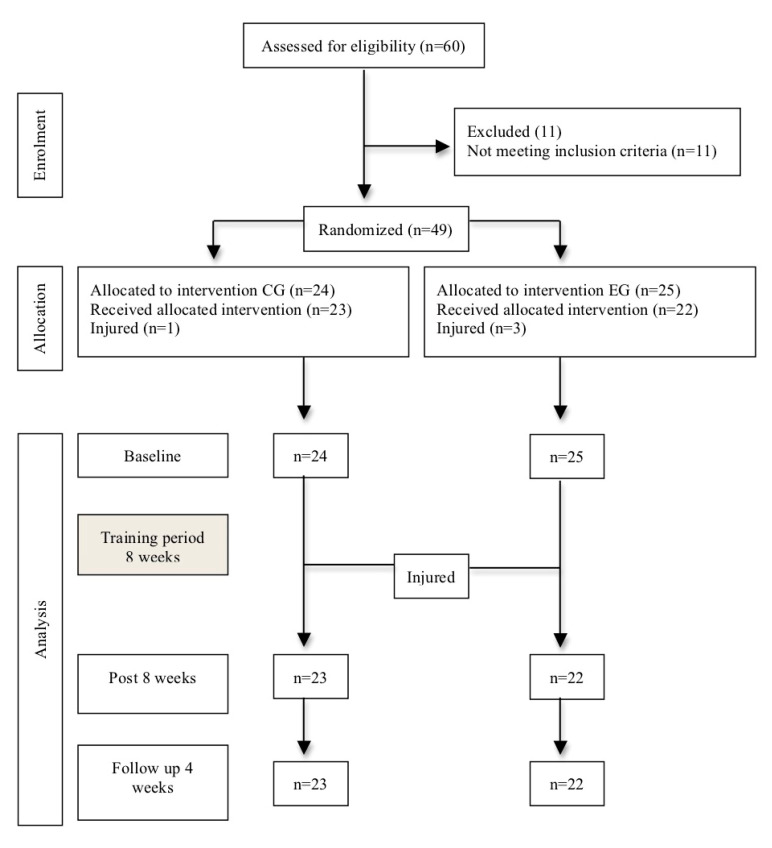
Consort diagram demonstrating participant flow through the study. Abbreviations: CG: control group; EG: experimental group.

Forty-nine subjects, all amateur male and female athletes, between 18 and 35 years of age, were recruited to participate in this study. They were athletes belonging to amateur football, volleyball and basketball teams who play each sport regularly. Subjects were recruited through announcements in sport facilities. Those subjects who did not present a body mass index (BMI) between 19 and 25, or who had suffered any lower limb pathology and/or any kind of cervical or lumbar pain process in the previous six months, were excluded (Figure 1).

All subjects were interviewed individually, where they were informed in detail of the risks and benefits, both verbally and in writing. Written informed consent was requested from all of them, both for participating in the study and for filming, as well as for the use of data and images for future research or presentations of the current study.

### 2.3. Randomization

Participants were assigned using a computer-generated table of random numbers to either an experimental or a control group in a proportion of 1:1. Assignments were made using sealed opaque envelopes, consecutively numbered and kept under lock. Finally, 25 subjects were assigned to the experimental group and 24 to the control group.

### 2.4. Outcomes

Height and weight were assessed by a T201-T4 Asimed adult height scale and a 100 g–130 kg precision digital weight scale (Tefal), respectively. Body mass index (BMI) was obtained by dividing the weight (kg) by the height squared (m^2^). To assess lower extremity, femur and tibia length, we used an inelastic measuring tape placed at the anterior superior iliac spine and the medial malleolus (lower extremity length), at the great trochanter and the lateral femoral condyle (femur length) and at the fibular head and medial malleolus (tibia length). Thigh, leg and arm diameter was measured using an inelastic measuring tape at the mid muscle belly.

Three measurements were made during the study. A first measurement was made after the individualized interviews and the signing of the informed consents, and prior to the intervention, where all the subjects performed three jumps, among which the highest one was selected. The second measurement took place the day after the eight-week intervention. Once the intervention was completed, both groups were told to maintain their regular training routine and the subjects in the experimental group were told not to perform any of the gluteal specific exercises for four weeks. After that period, subjects were called to carry out the third measurement. To measure the squat jump, the app ‘My Jump 2’^©^ (2016–18 Carlos Balsalobre-Fernández) was used on an iPhone 8 (Apple Inc., Cupertino, CA, USA). This app has been previously validated (ICC = 0.997) [19]. This app directly measures flight time and, indirectly, jump height, speed and power. The iPhone 8 (Apple Inc., Cupertino, CA, USA) was placed on a tripod facing the subject and 1.5 m away.

**Figure 2:**
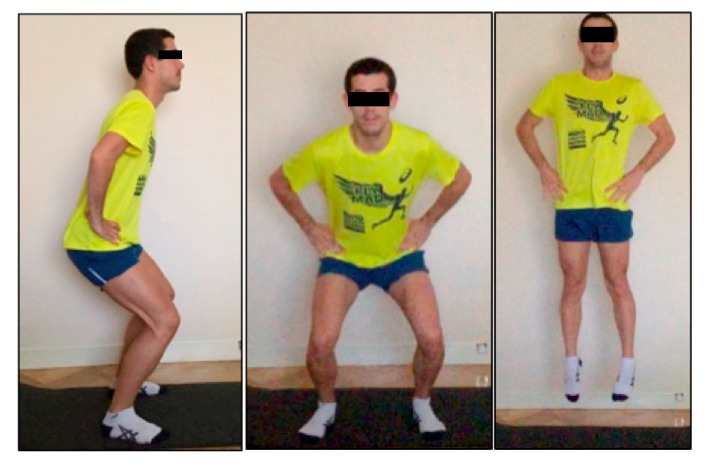
Squat jump position and execution.

Execution of the squat jump: Subjects performed a barefoot squat jump on a non-deformable surface. The starting position was standing with a ~90° knee flexion, ~100° hip flexion and tiptoes facing forward (Figure 2), as described elsewhere [20,21]. During all phases of the jump, the hands remain on the hips, in order to eliminate any influence of the arms during execution. In the take-off phase, subjects extend both knees and hips, and perform a plantar flexion until their toes leave the floor. When landing, subjects end in the same initial position. In order to minimize the effect of myofascial elastic energy storage, a three-second period maintaining the starting position was established before jumping, without a countermovement phase previous to initiating the jump [21]. Three jumps with a 30 s rest period were performed; the one with the greatest height was selected.

### 2.5. Intervention

**Figure 3:**
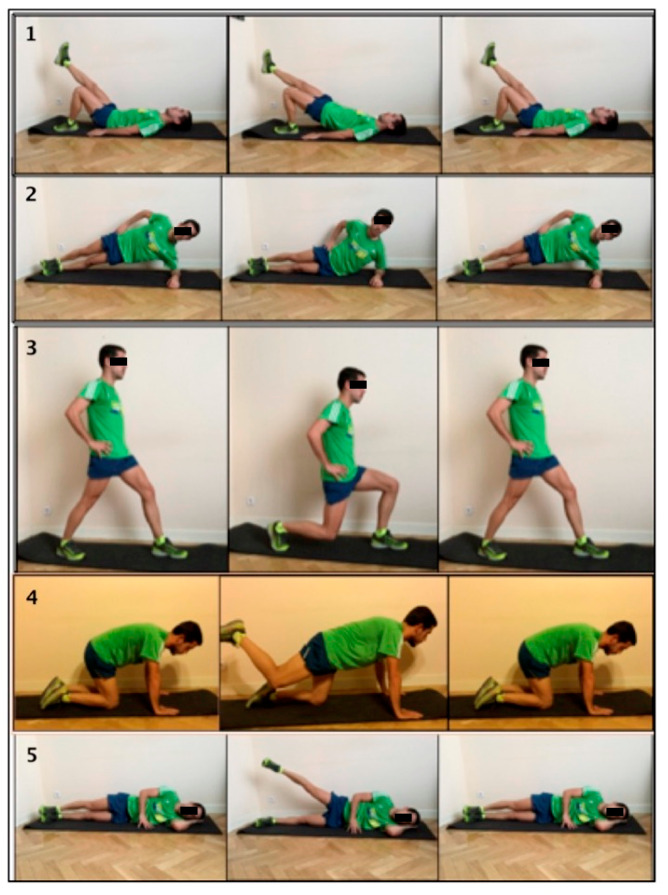
Gluteal strengthening exercises: (**1**) Shoulder bridge with elevation to one leg: make sure the bent knee does not turn medial or laterally; do not drop the contralateral hemipelvis either. (**2**) Lateral plank with two supports. (**3**) Lunge: forward knee must not be pushed out farther than the big toe. (**4**) Quadrupeia with three supports and knee bent: slight pelvic retroversion to inhibit erector muscles. (**5**) One leg elevation with trunk resting on the floor.

Subjects in the experimental group performed five exercises that have been shown to achieve moderate to high activation of the gluteus maximus and medium [22]. These exercises were performed three times a week for eight weeks, none of them with added load. In each session, a total of two sets of 12 repetitions each were carried out, with a 1 min rest time between sets [23]. The concentric and eccentric relationship movement was 1/3 [4] (Figure 3). In the first session, a researcher instructed the subjects in the experimental group on how to perform the exercises correctly. During the first week, this researcher did a face-to-face evaluation of the exercises performed. After the first week, a telephone follow-up was done.

Both groups were told to maintain their regular training routine and to note any change to their habitual load. Tracking of a subject’s activity in the experimental group was achieved by means of a diary (Annex 1). This diary had to be delivered at the end of the treatment period to a researcher.

### 2.6. Sample Size Calculation

Sample size was determined using MedCalc 16.4 software. A minimum sample size was used to determine whether there were significant differences between the control and intervention groups, with *β* values of 0.2 (80%) and alpha error of 0.05 (5%). Data provided by Haddas et al. [24] were used to obtain statistically significant differences between the groups, with a participation of at least 18 subjects per group, which made up for a total of 36 subjects. Bearing in mind that the expected dropout rate was 10%, a total of at least 40 subjects would be necessary to carry out the study. Finally, a total of 45 subjects were selected to participate in the study.

### 2.7. Statistical Analysis

Data were analyzed using SPSS v.22 software for Windows. All statistical tests were carried out considering a 95% confidence interval (*p*-value < 0.05). First, the Kolmogorov–Smirnov test was used with the Lilliefors significance correction to assess data normality, considering all subjects as a whole. Secondly, the Shapiro–Wilks test was performed on each group independently. Four dependent variables were incorporated into the design of the study: jump height, flight time, speed and power. A data descriptive analysis was carried out using means and their corresponding standard deviations for the variables age, size, weight, BMI, lower limb length, femur length, tibia length, thigh diameter, jump height, flight time, speed, power and sleep hours. Percentages were used for the remaining variables. The design used to find out whether differences in the analyzed variables were due to treatments, times or their interaction was a general linear model of repeated measures. Sphericity was tested using the Mauchly test, and in those cases where it was not met, the Greenhouse–Geisser correction was used. Adjustment for multiple comparisons was done using the Bonferroni correction, and effect size was estimated with the Eta-squared parameter (η^2^). Relative increase in the variables between groups was included by means of the formula = 100 * (* means times) (Media post3months-Mediapre)/Mediapre.

## 3. Results

Forty-five amateur athletes finished the intervention, of which 57.8% were female and 42.2% were male, with an average age of 27.33 ± 3.55; weight 63.04 ± 9.24 kg and BMI 21.52 ± 1.88. Homogeneity was found between the variance of differences between pairs of measures, so sphericity assumption was met. Descriptive sample data are collected in Table 1. During the study period, four subjects (one from the control group and three from the experimental group) were injured due to their regular sport practice and had to leave the study before finishing it.

### 3.1. Jump Height

Regarding the variable jump height, statistically significant differences between groups were found in the interaction of treatments and times (F_(2,86)_ = 5.174, *p* < 0.014; η^2^ = 0.10), between jumps 1 and 2 [3.77 (0.50, 7.04); *p* < 0.021]. The effect size is considered to be small. Differences between jumps 1 and 2 were found within the groups themselves, favoring the experimental group (Table 2). Jump increase in the experimental group (17.15%) is expressed as a significant improvement compared to the control group (3.09%) and to itself.

### 3.2. Fight Time

Statistically significant differences between groups were found in the variable flight time regarding the interaction of treatments and times (F_(2,86)_ = 8.835, *p* < 0.001; η^2^ = 0.17), between flight time 1 and flight time 2 [3.23 (−14.24, 30.72); *p* < 0.03]. The effect size is considered to be small. Differences between flight time 1 and flight time 2 were found within the groups themselves, favoring the experimental group (Table 3). Flight time increase in the experimental group (−7.98%) is expressed as a significant improvement compared to the control group (3.52%) and to itself.

### 3.3. Speed

Statistically significant differences between groups were found in the variable speed regarding the interaction of treatments and times (F_(2,86)_ = 5.130, *p* < 0.008; η^2^ = 0.12), between speed 1 and speed 2 [0.05 (0.01, 0.10); *p* < 0.018]. The effect size is considered to be small. Differences between speed 1 and speed 2 were found within the groups, favoring the experimental group (Table 4). Jump speed increase in the experimental group (1.96%) is expressed as a significant improvement compared to the control group (1.83%) and to itself.

### 3.4. Power

Statistically significant differences between groups were found in the variable power regarding the interaction of treatments and times (F_(2,86)_ = 3.733, *p* < 0.028; η^2^ = 0.08), between power 1 and power 2 [28.33 (8.03, 48.64); *p* < 0.008]. The effect size is considered to be small. Differences between speed 1 and power 2 were found within groups, favoring the experimental group (Table 5).

Increase in jump power in the experimental group (4.43%) is expressed as a significant improvement compared to the control group (0.32%) and to itself.

## 4. Discussion

The purpose of this study was to investigate the effect of an eight-week course of gluteal muscles specific training on the squat jump. The main findings in this study demonstrate that such a program has a significant effect on squat jump capacity, increasing jump height, flight time, speed and power, compared to a control group. This may be due to the fact that the pelvis stability comes mainly from the lumbo-pelvic-hip complex, specifically through gluteal muscle activation. In addition, the *gluteus maximus* is the main hip extensor and external rotator muscle, and the *gluteus medius* is the main frontal plane pelvic stabilizer [1], so a gluteal-specific training regime should increase hip stability in the frontal and sagittal planes and increase the hip extensor moment, thereby contributing to improvements in the squat jump.

Our data on jump height and flight time revealed a significant increase for the intervention group compared to the control group. Although we found no studies that used a gluteal-specific training regime to asses changes in squat jump performance, our results are in concordance with those of Crow et al. [25], Healy and Harrison [26], and Harrison and McCabe [27], which show incremental increases in jump height [25] and in flight time [26,27] after a warm-up exercise aimed at the gluteus muscles in trained men and women, performing drop jumps, single drop jumps and countermovement jumps. This result may be due to increased joint stability of the knee during the final take-off of the squat jump, which could provide a more efficient transfer of force through the joint [28]. Similar results were obtained after facilitating the activation of the erector spinae before a squat jump [3]. However, Comyns et al. [29] published data contradicting our results, finding that low load gluteal activation exercises decrease flight time, after 30 s and after 6 min post-warm-up. This discrepancy may be due to fatigue; while Comyns et al. [29] used a low load gluteal warm-up before the testing, we used a general warm-up. In addition, in our study the participants were enrolled to an 8-week gluteal exercise training that could prevent the fast fatigue of the gluteal muscles. These results support the idea that the gluteal muscles play a major role in squat jumps. In this study, we measured a squat jump using both legs, so the frontal plane pelvic stability roll of the gluteal muscles is probably not a major issue, the principal roll of the gluteal muscles being the hip extension. Many sports situations require a single leg jump, where the frontal plane pelvic stability role may be the determinant.

Jump speed and power showed a significant increase following the intervention compared to the control group. Our results are in agreement with those by Mike et al. [4], who proved there was a significant increase in jump power after completing a four-week closed kinetic chain eccentric exercise program “using a plate-loaded barbell Smith squat exercise” with loads of 80–85%1RM. Pupo et al. [30] concluded that the height reached in a squat jump is associated with maximum speed and maximum force, since this type of jump depends only on muscle action during the concentric phase. Even though some studies have associated jump power to strength and speed training [30,31], our results show that pure ballistic training is not necessary. The stabilizing role of the gluteal musculature of the pelvis is mainly eccentric, whereas the impulse phase is concentric, both being antagonistic training forms. Battaglia et al. [32] suggested that the specificity of training in jump sports may promote neuromuscular adaptations that influence the performance. Future studies that assess both roles and their relationship with the jump can facilitate the choice in the type of training of the gluteal muscles.

Some limitations of this study should be considered when interpreting the results; first, the lack of homogeneity of the type of sport subject’s practice and individual fitness could be a source of bias, because some sports may have a greater impact on gluteal muscles, hampering the generalizability of these results. Second, the amount of training time was not equalized for the control and experimental group, so it is possible that results were due to more training rather than a benefit of specific gluteal muscle training. Third, although all subjects were asked to maintain their habitual practice, there was no controlling of the amount of exercise outside the study. Finally, although ‘My Jump 2’ has been validated [19], the results depend on the human eye when selecting the take-off and landing photographs, as opposed to force platforms (the gold standard method), which perform a direct measurement of take-off and landing.

Future studies assessing the impact of the stability of the pelvis on the frontal plane during a single leg jump, and whether a training regimen aimed at the gluteal muscles could affect the frontal plane stability of the pelvis and improve jumping ability, are needed.

## 5. Conclusions

An eight-week program specifically aimed to target the gluteal muscles improves squat jump performance. Our results inform physical conditioning professionals and athletes who perform vertical jumps about the efficacy of gluteal muscle strengthening programs in their training regimen. Nevertheless, additional studies are necessary for specific gluteal muscle training, combined with other types of training (strengthening plus speed) in order to widen our knowledge on the improvement of vertical jump performance.

## Figures and Tables

**Table 1:** Participants demographics and outcome measures at baseline.

Variables	Total Group *N* = 45	Control Group *N* = 23	Experimental Group *N* = 22	*p*
Sex/F–M in %	26–19 (57.8%–42.2%)	14–9 (60.8%–29.2%)	12–10 (54.6%–45.4%)	0.767
Age/Years/Max.-min. *	27.33 (3.55) 19–35	27.43 (3.89) 19–33	27.23 (3.35) 24–35	0.847
Size/m *	1.70 (0.09)	1.70 (0.08)	1.70 (0.09)	0.984
Weight/kg *	63.04 (9.24)	63.35 (9.00)	62.71 (9.60)	0.821
BMI/kg:m^2^ *	21.52 (1.88)	21.63 (1.83)	21.41 (1.96)	0.696
Length mmii/cm *	80.16 (4.73)	79.76 (4.64)	80.59 (4.90)	0.563
Femur length/cm *	40.56 (3.19)	40.19 (3.37)	40.95 (3.03)	0.432
Tibia length/cm *	38.27 (2.70)	38.15 (2.56)	38.40(2.89)	0.754
Thigh diameter/*d* = 2r *	50.34 (4.37)	50.28 (2.21)	50.41 (5.91)	0.919
Triceps diameter/*d* = 2r *	35.46 (3.10)	35.32 (2.30)	35.60 (3.81)	0.771
SJ pre/cm *	24.05 (10.37)	22.61 (10.20)	25.56 (10.58)	0.347
Speed pre/m:s *	1.05 (0.26)	1.02 (0.26)	1.09 (0.25)	0.387
Power pre/W *	415.85 (97.97)	410.67 (91.71)	421.27 (106.02)	0.721
FT pre/ms *	467.04 (106.69)	418.43 (108.58)	517.86 (78.78)	0.405
Smoker/yes-no in %	3–42 (6.7%–93.3%)	1–22 (4.3%–95.7%)	2–20 (9%–91%)	
Sport/no. in %:				
Football Basketball Volleyball	20 (44.4%) 12 (26.7%) 13 (28.9%)	10 (43.5%) 6 (26.1%) 7 (30.4%)	10 (45.6%) 6 (27.2%) 6 (27.2%)	
Sleep hours/hours *	7.11 (0.82)	7.13 (0.78)	7.09 (0.88)	

Abbreviations: F: female; M: male; SD: standard deviation; Max.: maximum; Min.: minimum; SJ: squat jump; FT: flight time; BMI: body mass index; * normal variable values are mean ± SD (95% confidence interval).

**Table 2:** Jump height intra- and inter-group differences.

Group *	Pre Intervention	Post Intervention	Follow-Up	% RC
Control (cm)	22.61 (10.20)	23.41 (8.86)	23.31 (9.09)	3.09
Experimental (cm)	25.56 (10.58)	30.14 (8.32)	26.43 (10.57)	17.15
**Within-Group Difference in Change Score ^†,‡^**				
**Time Interaction Per Group**	F_(2,86)_ = 5.174, *p* = 0.014; η^2^ = 0.10			
**Intra Group Time Differences**				
Control/pre-post; pre-follow-up		−0.80 (−3.55, 1.94)	−0.70 (−2.29, 0.89)	
Experimental/pre-post; pre-follow-up		−4.58 (−7.38, −1.77) ~	−0.87 (−2.48, 0.75)	
**Inter group time differences ^‡^** pre-post; pre-follow-up		3.77 (0.50, 7.04) ~	0.17 (−1.67, 2.01)	

Abbreviations: SJ: squat jump; * means and standard deviation; ^†^ compared to pretreatment; ^‡^ mean difference (95% confidence interval); ~ statistically significant differences (*p* < 0.05); η^2^ = Eta squared. Effect Size; %RC = percentage relative change = (V1–V2)/V2 × 100. Bold: refers to another analysis.

**Table 3:** Flight time intra- and inter-group differences.

Group *	Pre Intervention		Post Intervention	Follow-Up	% RC
Control (ms)	418.43 (108.58)		431.69 (93.39)	433.17 (98.15)	3.52
Experimental (ms)	517.86 (76.78)		579.77 (93.39)	531.72 (43.98)	7.98
**Within-Group Difference in Change Score ^†,‡^**					
**Time Interaction Per Group**		F_(2,86)_ = 8.835, *p* < 0.001; η^2^ = 0.17			
**Intra Group Time Differences**					
Control/pre-post; pre-follow-up			−13.26 (−28.36, 1.84)	−14.73 (−54.91, 25.43)	
Experimental/pre-post; pre-follow-up			−16.50 (−31.94, −1.05) ~	−14.59 (−56.66, −26.59)	
**Inter group time differences ^‡^** pre-post; pre-follow-up			3.23 (−14.24,30.72) ~	−0.14 (1.75, 52.02)	

Abbreviations: FT: flight time; * means and standard deviation; ^†^ compared to pretreatment; ^‡^ mean difference (95% confidence interval); ~ statistically significant differences (*p* < 0.05); η^2^ = Eta squared. Effect Size; %RC = percentage relative change = (V1−V2)/V2 × 100. Bold: refers to another analysis.

**Table 4:** Speed intra- and inter-group differences.

Group *	Pre Intervention	Post Intervention	Follow-Up	% RC
Control (m/s)	1.02 (0.26)	1.05 (0.22)	1.04 (0.23)	1.83
Experimental (m/s)	1.09 (0.25)	1.17 (0.27)	1.11 (0.25)	1.96
**Within-Group Difference in Change Score ^†,‡^**				
**Time Interaction Per Group**		F_(2,86)_ = 5.130, *p* = 0.008; η^2^ = 0.12		
**Intra Group Time Differences**				
Control/pre-post; pre-follow-up		−0.02 (−0.06, 0.01)	−0.02 (−0.06, 0.01)	
Experimental/pre-post; pre-follow-up		−0.08 (−0.12, −0.04) ^§^	−0.02 (−0.05, 0.01)	
**Inter group time differences ^‡^** pre-post; pre-follow-up		0.05 (0.01, 0.10) *~*	−0.00 (−0.04, 0.03)	

* Means and standard deviation; ^†^ compared to pretreatment; ^‡^ mean difference (95% confidence interval); ^§^ statistically significant differences (*p* < 0.01); ~ statistically significant differences (*p* < 0.05); η^2^ = Eta squared. Effect Size; %RC = percentage relative change = (V1−V2)/V2 × 100. Bold: refers to another analysis.

**Table 5:** Power intra- and inter-group differences.

Group *	Pre Intervention		Post Intervention	Follow-Up	%RC
Control (watts)	410.67 (91.71)		416.42 (96.85)	412.00 (83.90)	0.32
Experimental (watts)	421.27 (106.02)		455.35 (97.50)	426.77 (103.17)	4.43
**Within-Group Difference in Change Score ^†,‡^**					
**Time Interaction Per Group**		F_(2,86)_ = 3.733, *p* = 0.028; η^2^ = 0.08			
**Intra Group Time Differences**					
Control/pre-post; pre-follow-up			−5.74 (−23.38, 11.79)	−1.33 (−19.59, −16.93)	
Experimental/pre-post; pre-follow-up			−34.08 (−52.02, −16.14) ^§^	−5.50 (−24.18, 13.17)	
**Inter group time differences ^‡^** pre-post; pre-follow-up			28.33 (8.03, 48.64) ~	4.17 (−16.97, 25.31)	

* Means and standard deviation; ^†^ compared to pretreatment; ^‡^ mean difference (95% confidence interval); ^§^ statistically significant differences (*p* < 0.01); ~ statistically significant differences (*p* < 0.05); η^2^ = Eta squared. Effect Size; %RC = percentage relative change = (V1−V2)/V2 × 100. Bold: refers to another analysis.
